# Chemical Characterization of Volatile Terpenoids in *Teucrium polium* L. Essential Oil Evaluated by GC‐MS/FID and Its Associated Biological Properties

**DOI:** 10.1002/cbdv.71480

**Published:** 2026-07-15

**Authors:** Fatima Zahra Sadiki, Souhail Channaoui, Aziz Bouymajane, Mostafa El Idrissi, Ali Amechrouq, Mohammed Sbiti, Francesco Cacciola, Mohamed Chabbi

**Affiliations:** ^1^ Laboratory of Molecular Chemistry and Natural Substances Department of Chemistry Faculty of Sciences Moulay Ismail University Meknes Morocco; ^2^ Oasis System Research Unit Regional Center of Agricultural Research of Errachidia National Institute of Agricultural Research Rabat Morocco; ^3^ Biology, Environment and Health Team Faculty of Sciences and Techniques of Errachidia Moulay Ismail University Morocco; ^4^ Laboratory of Microbiology Military Hospital Moulay Ismail Meknes Morocco; ^5^ Messina Institute of Technology c/o Department of Chemical, Biological, Pharmaceutical and Environmental Sciences University of Messina Messina Italy; ^6^ Laboratory of Physical‐Chemistry of Materials Natural Substances and Environment Department of Chemistry Faculty of Sciences and Techniques Abdelmalek Essâadi University Tangier Morocco

**Keywords:** antibacterial, antioxidant, GC‐MS/FID, insecticidal, *Teucrium polium* L

## Abstract

The study aimed to characterize the chemical composition, and evaluate the antioxidant, antibacterial, and insecticidal activities of *Teucrium polium* essential oil (TP‐EO) collected from the northeast of Morocco. The antioxidant activity was carried out using ABTS and DPPH radical scavenging assays, whereas the insecticidal properties of TP‐EO were evaluated against *Sitophilus granarius* and Tribolium confusum. In addition, the antibacterial effects of TP‐EO were tested against a broad spectrum of Gram‐positive and Gram‐negative bacterial strains. GC‐MS/FID analyses allowed to identify a total of 64 volatile compounds in TP‐EO. The major constituents were β‐eudesmol (10.91%), β‐pinene (10.63%), α‐eudesmol acetate (9.22%), and α‐pinene (7.69%). Furthermore, TP‐EO exhibited remarkable bactericidal effects against all tested bacteria except *Klebsiella pneumoniae* 1 and *Proteus penner*, with the MIC values ranging from 2.5 ± 0.1 to 20 ± 0.1 µL/mL. In addition, TP‐EO displayed notable insecticidal effects against both Sitophilus granarius and Tribolium confusum, and moderate antioxidant activity. Therefore, TP‐EO could serve as a valuable, natural alternative in food safety, organic farming, and pest management, providing a sustainable option to reduce reliance on synthetic chemicals.

## Graphical abstract

GC–MS/FID profiling of *Teucrium polium* L. essential oil reveals a terpenoid‐rich composition with promising antimicrobial, antioxidant, and insecticidal activities.

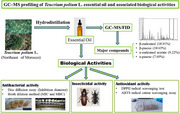

## 1 Introduction

Medicinal plants have long formed the basis of therapeutic remedies, largely thanks to their high concentration of secondary metabolites, including flavonoids, terpenoids, alkaloids, and phenolic compounds [1]. These compounds are often associated with antimicrobial, antioxidant, and insecticidal properties [2]. The increasing prevalence of antibiotic‐resistant bacteria, the health risks associated with oxidative stress and the environmental and health concerns arising from the use of synthetic insecticides have prompted a global shift toward natural plant‐derived alternatives [3].

*Teucrium polium* L. is a perennial plant belonging to the *Lamiaceae* family. It is widely distributed across the Mediterranean basin, the Middle East and parts of North Africa. It has traditionally been used to treat ailments such as diabetes, gastrointestinal disorders, infections, and inflammation [4, 5, 6, 7, 8]. The plant produces a variety of biologically active secondary metabolites, including essential oils (EOs), diterpenoids, and phenolic compounds [8, 9, 10].

Essential oils (EOs) derived from *T. polium* have received a lot of attention because of their important biological properties, especially their antibacterial and antioxidant effects. Studies have demonstrated the inhibitory effects of the EO against various Gram‐positive and Gram‐negative bacteria, including *Staphylococcus aureus*, *Escherichia coli*, and *Pseudomonas aeruginosa* [11, 12, 13]. In addition to its antimicrobial potential, *T. polium* EO (TP‐EO) demonstrates antioxidant activity, which is particularly important given the significant impact of oxidative stress on ageing and the development of chronic diseases, including neurodegenerative and cardiovascular disorders. Oxidative stress arises from an imbalance between the production of reactive oxygen species (ROS) and the body's ability to detoxify them, resulting in cellular damage. In this context, natural antioxidants from medicinal plants are increasingly valued for their ability to scavenge free radicals and reinforce the body's defence systems [14, 15].

Moreover, EOs have shown promise as natural insecticides. Insects, like *Sitophilus granaries* and *Tribolium confusum* cause significant post‐harvest damage and are often controlled using synthetic chemicals. However, the use of these conventional pesticides can result in resistance developing in the insects, as well as causing environmental pollution and leaving harmful residues in food [16]. Botanical insecticides derived from essential oils provide a more environmentally friendly alternative, as demonstrated by recent studies evaluating the insecticidal activity of medicinal plant species [17, 18, 19]. A study by Benali et al. [20] on *T. polium* subsp. *polium* from northern Morocco revealed an EO composition containing high levels of β‐pinene and germacrene D, demonstrating notable antioxidant and antibacterial properties against multiple strains, including *Bacillus subtilis* and *Proteus mirabilis*. However, the oil exhibited limited antifungal activity against *Candida albicans*. These findings highlight the importance of regional studies, as the biological activities and chemical compositions of EO can vary significantly depending on geographical and ecological conditions.

Despite these promising insights, there is limited data on the TP‐EO in arid and semi‐arid ecosystems, such as those in the northeast of Morocco, where environmental stressors may influence the synthesis of bioactive compounds. In this context, the essential oil of *T. polium* collected from the northeastern region of Morocco was investigated, revealing a distinct chemical profile compared to previously reported data. Such variability may also affect the biological activities of the essential oil, highlighting the importance of investigating both its chemical composition and associated bioactivities. The present study, therefore aims to (i) determine the chemical composition of TP‐EO from this under‐explored region using gas chromatography‐mass spectrometry (GC‐MS); and (ii) evaluate its antioxidant, antibacterial, and insecticidal activities. The results are expected to contribute to the promotion of Moroccan medicinal flora and the development of natural alternatives for pharmaceutical and agricultural applications.

## 2 Results and Discussion

### 2.1 Chemical Composition of TP‐EO

**FIGURE 1:**
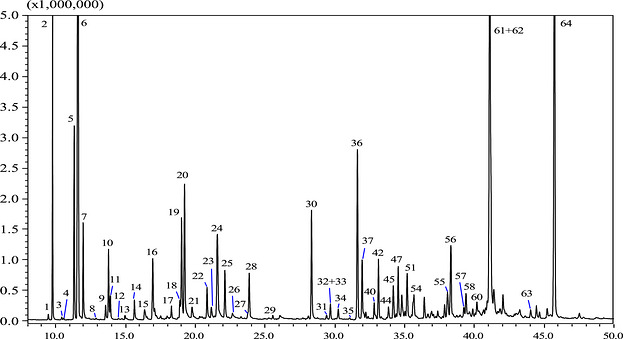
GC‐MS chromatogram of TP‐EO.

GC‐MS/FID analysis of our essential oil revealed a chemical profile dominated by monoterpenes and oxygenated sesquiterpenes (Figure 1). The major constituents identified are β‐eudesmol (10.91%), β‐pinene (10.63%), α‐eudesmol acetate (9.22%), α‐pinene (7.69%), (*E*)‐caryophyllene (4.24%), sabinene (4.17%), myrtenal (3.70%), (*E*)‐verbenol (3.68%), and (*E*)‐pinocarveol (3.00%) (Table 1). This composition suggests a chemotype rich in oxygenated sesquiterpenes and bicyclic monoterpenes. The chemical profile obtained in the present study differs from that reported for TP‐EO collected in the province of Midelt, Morocco, where δ‐3‐carene (16.49%), γ‐Muurolene (14.03%), and α‐pinene (9.94%) were the major compounds, indicating a chemotype distinct from that observed in the eastern region of the country [21]. In Jordan, a study conducted on *T. polium* highlighted the dominance of 8‐cedren‐13‐ol (24.80%), β‐caryophyllene (8.70%), germacrene D (6.83%), also a different composition from our essential oil [22]. Djabou et al. indicated that the essential oil of *T. polium* is rich in α‐pinene (33.2%), α‐thujene (8.1%), and terpinen‐4‐ol (6.6%) [23]. In Croatia, Bezić et al. identified β‐caryophyllene (52%) and germacrene D (8.7%) as major compounds in essential oils from the aerial part of *T. polium* [24]. Furthermore, Vahdani et al. reported a dominance of limonene (37.70%) and 2,4 di‐tetra‐butylphenol (10.81%) in the essential oil of *T. polium* from Iran [25], illustrating yet another different chemotype. A recent work confirms this marked chemical variability, on the essential oil of *T. polium* L. collected from Jericho, the lowest point on Earth, where E‐nerolidol (27.11%), geranyl acetone (23.26%), germacrene D (19.08%), β‐caryophyllene (17.78%), are the major compounds [26]. Overall, these results highlight the existence of several chemotypes of *T. polium* across the Mediterranean basin and the Middle East. These differences could be attributed to the geographical origin, climatic conditions, and phenological stage of the plant at the time of harvest.

**TABLE 1:** Identity of terpene and terpenoid compounds in TP‐EO.

ID	Compounds	MS Match	LRI exp	LRI ref	TP‐EO	
					%	RSD
1	α‐thujene	97	927	927	0.13	1.29
2	α‐pinene	98	935	933	7.69	0.61
3	Camphene	92	952	953	0.06	0.00
4	Thuja‐2,4(10)‐diene	94	956	953	0.11	1.37
5	Sabinene	98	975	972	4.17	0.50
6	β‐pinene	97	981	978	10.63	0.52
7	Myrcene	98	990	991	2.00	0.40
8	δ‐3‐Carene	89	1012	1009	0.03	1.73
9	*p*‐cymene	98	1027	1025	0.37	0.54
10	Limonene	98	1032	1030	1.72	0.58
11	Eucalyptol	92	1035	1032	0.48	1.26
12	(*E*)‐β‐ocimene	91	1047	1046	0.05	1.08
13	2‐methylbutyl‐butanoate	97	1058	1056	0.07	0.86
14	(*Z*)‐Sabinene hydrate	94	1073	1069	0.46	0.57
15	(*E*)‐Linalool oxide	92	1089	1086	0.34	0.51
16	Linalool	98	1102	1101	1.45	0.42
17	α‐campholenal	95	1130	1125	0.36	0.56
18	Nopinone	95	1143	1139	0.56	1.53
19	(*E*)‐pinocarveol	97	1146	1141	3.00	0.60
20	(*E*)‐verbenol	98	1150	1145	3.68	0.47
21	Sabina ketone	92	1162	1157	0.46	0.22
22	Terpinen‐4‐ol	93	1185	1184	0.80	0.94
23	*p*‐cymen‐8‐ol	90	1192	1189	0.44	1.72
24	Myrtenal	94	1201	1197	3.70	0.66
25	Verbenone	98	1213	1208	1.49	0.81
26	(*E*)‐carveol	94	1225	1223	0.40	5.68
27	Cuminaldehyde	92	1248	1243	0.23	1.94
28	Linalyl acetate	97	1251	1250	0.97	8.64
29	Bornyl acetate	95	1288	1285	0.08	0.68
30	α‐terpinyl acetate	98	1350	1349	2.29	0.66
31	α‐ylangene	88	1374	1371	0.11	0.88
32	(*E*)‐geranyl acetate	90	1379	1380	0.38	0.85
33	α‐copaene	94	1380	1375		
34	β‐elemene	94	1394	1390	0.31	3.65
35	β‐maaliene	86	1413	1415	0.05	11.16
36	(*E*)‐caryophyllene	98	1426	1424	4.24	0.47
37	γ‐Elemene	92	1434	1432	1.43	0.21
38	α‐(E)‐bergamotene	90	1437	1432	0.11	1.83
39	α‐Guaiene	90	1440	1438	0.16	0.64
40	(E)‐β‐farnesene	97	1455	1452	0.41	0.37
41	Sesquisabinene	88	1457	1455	0.08	3.77
42	α‐Humulene	97	1460	1454	1.43	0.57
43	9‐epi‐(*E*)‐caryophyllene	92	1467	1464	0.08	2.46
44	Selina‐4,11‐diene	95	1479	1476	0.39	0.82
45	Germacrene D	95	1487	1480	0.89	0.79
46	(Z)‐β‐guaiene	90	1492	1498	0.16	1.56
47	β‐Selinene	96	1496	1492	1.49	0.54
48	Valencene	88	1497	1492	0.11	1.83
49	epi‐Cubebol	92	1502	1498	0.77	0.79
50	α‐Bulnesene	90	1508	1505	0.18	0.63
51	β‐Bisabolene	95	1512	1508	1.18	0.54
52	γ‐cadinene	90	1519	1512	0.10	1.00
53	Cubebol	95	1522	1519	0.42	1.44
54	δ‐cadinene	94	1525	1518	0.50	0.23
55	Spathulenol	93	1580	1576	0.92	0.66
56	Caryophyllene oxide	96	1591	1587	1.75	0.46
57	5‐epi‐7‐epi‐α‐Eudesmol	93	1615	1610	0.34	0.00
58	Humulene epoxide II	91	1619	1613	0.79	0.26
59	γ‐eudesmol	94	1632	1632	0.27	4.74
60	Agarospirol	89	1640	1646	0.46	2.85
61	β‐eudesmol	90	1666	1656	10.91	0.10
62	Oplopanone	91	1743	1738	0.23	0.00
63	α‐eudesmol acetate	90	1791	1793	9.22	0.52
	Not Identified				11.91	2.79
	Total				100	—

Abbreviations: MS Match: database spectral similarity; LRI exp: experimental LRI; LRI ref: reference LRI. The volatile compounds are expressed in % values. RSD is relative standard deviation of the replicated measurements.

### 2.2 Antioxidant Activity

The antioxidant activity of the TP‐EO was evaluated using DPPH superoxide anion radical (O_2_^•−^) scavenging method and ABTS radical scavenging assay, and the results are presented as IC_50_ values in Table 2. The IC_50_ value indicates the concentration required to inhibit 50% of free radicals, so lower IC_50_ values correspond to higher antioxidant capacity.

**TABLE 2:** Antioxidant power of TP‐EO expressed in IC_50._

Test	IC_50_ (mg /mL)
DPPH	7.60 ± 0.2
ABTS	1.23 ± 0.1
Ascorbic acid	0.5 ± 0.1
Gallic acid	0.2 ± 0.1

TP‐EO exhibited moderate antioxidant activity, with IC_50_ values of 7.60 ± 0.2 and 1.23 ± 0.1 mg/mL for the DPPH and ABTS assays, respectively. These values are significantly higher than those obtained for standard antioxidants such as ascorbic acid (0.5 ± 0.1 mg/mL) and gallic acid (0.2 ± 0.1 mg/mL), indicating noteworthy antioxidant potential despite being lower. The stronger activity observed in the ABTS assay compared to the DPPH assay may be due to the EO's components having different solubility and reactivity toward hydrophilic and lipophilic radicals.

These results are consistent with those of previous studies on the antioxidant properties of *Teucrium* species. These species are known to contain a variety of bioactive compounds, such as flavonoids, terpenoids, and phenolic constituents [20, 27, 28, 29, 30]. Chabane et al. [10] reported the TP‐EO subsp. *capitatum* exhibited moderate antioxidant activity, as demonstrated by its effectiveness in the DPPH radical scavenging and β‐carotene bleaching assays. Similarly, Benali et al. [20] investigated the TP‐EO subsp. polium, reporting an IC_50_ value of 208.33 ± 3.51 µg/mL in the DPPH assay and a reducing power of 1.32 ± 0.1 mg AAE/g in the FRAP assay. The chemical composition of the oil was found to comprise mainly β‐pinene, germacrene D, and spathulenol, which supports the hypothesis that monoterpenes and sesquiterpenes play a critical role in the antioxidant activity of TP‐EO. Despite its lower potency compared to pure standards, TP‐EO may still be valuable as a natural antioxidant in pharmaceutical and food applications, where mild, broad‐spectrum antioxidant activity is beneficial.

### 2.3 Insecticidal Activity

**FIGURE 2:**
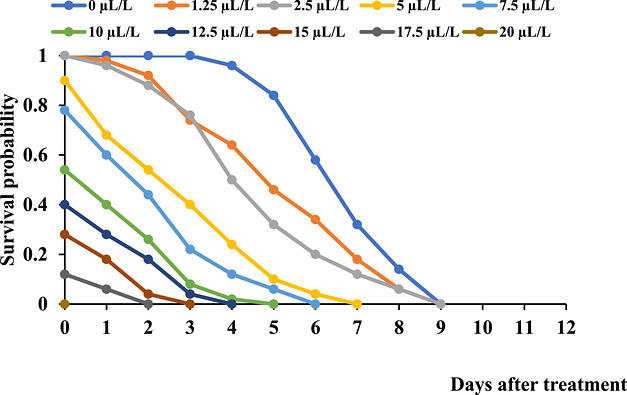
Survival of *S. granaries* adults exposed to TP‐EO.

**FIGURE 3:**
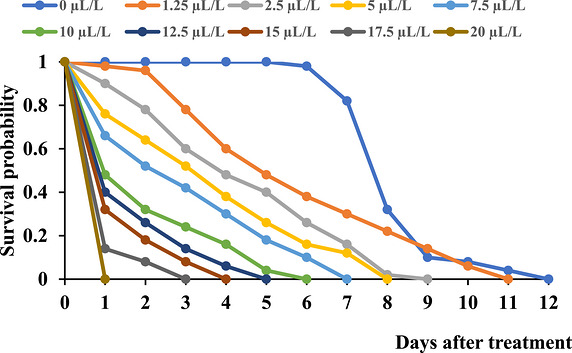
Survival of *T.confusum* adults exposed to TP‐EO.

The insecticidal efficacy of TP‐EO was evaluated in relation to adult *Sitophilus granarius* and *Tribolium confusum* insects. The results demonstrate strong dose‐ and time‐dependent toxicity for both species (Figures 2 and 3). Higher EO concentrations (15–20 µL/L) resulted in the complete mortality of both species within 2–6 days, while lower concentrations caused significant reductions in survival probability over time.

Toxicological analysis (Table 3) revealed that *S. granarius* was more susceptible to TP‐EO than *T. confusum*. After three days of exposure, the LC_50_ for *S. granarius* was 5.446 µL/L (95% CI: 3.881‐7.089), compared to 8.17  µL/L (2.207–5.559) for *T. confusum*. This trend was consistent across all time points. On Day 1, the LC_50_ for *S. granarius* was 10.371 µL/L (8.536–12.203), while that for T*. confusum* was significantly higher at 8.685 µL/L (6.535–11.157), indicating a slower onset of toxicity in the latter. The probit slopes decreased over time (from 4.99 to 2.96 for *S. granarius* and from 2.91 to 2.01 for *T. confusum*), demonstrating that the dose‐response relationship became progressively shallower as successive observations were dominated by survivors with greater tolerance. All calculated chi‐squared values remained below the tabulated chi‐squared (χ^2^) value for 2 degrees of freedom (df) at 5.991, indicating a satisfactory probit fit [31].

**TABLE 3:** Toxicity parameters of the TP‐EO recorded against *S. granarius* and *T. confusum* adults (µL/L).

	Days after treatment	Slope ± SE(1)	Calculated χ^2^	χ2 (0, 05; 2)	CL50 (µL.L^−1^)(2) [Confidence Interval]	CL99 (µL.L^−1^)(2) [Confidence Interval]
*S. granarius*	1	4993 ± 1024	1652	5991	10,371 [8536 – 12,203]	30,322 [22,138 – 60,806]
	2	3211 ± 0676	1359		7794 [5842 – 9957]	41,320 [25,087 ‐ 129,080]
	3	2958 ± 0562	2756		5446 [3,881 – 7,089]	33,303 [20,504 – 89,949]
*T.confusum*	1	2,905 ± 0,585	3401	5991	8685 [6535 − 11,157]	54,882 [32,065 − 179,431]
	2	2434 ± 0516	0984		6412 [4443 − 686]	57,924 [30,183 − 261,164]
	3	2007 ± 0447	3049		3,871 [2,207 – 5,559]	55,842 [26,453 – 339,652]

Furthermore, the LC_99_ values also indicate that TP‐EO is more toxic to *S. granarius*. After three days, the LC_99_ value for *S. granarius* was 33.303 µL/L (20.504–89.949), whereas *T. confusum* required a much higher concentration of 55.842 µL/L (26.435–339.652) to achieve 99% mortality. These findings clearly demonstrate that, *S. granarius* is more sensitive to TP‐EO and responds more rapidly to treatment.

This differential sensitivity may be attributed to species‐specific physiological and biochemical factors, such as variations in the activity of detoxification enzymes, cuticular permeability, or respiratory rate, which influence the penetration and efficacy of essential oils [32, 33]. The stronger response observed in *S. granarius* is consistent with previous studies that have reported the high sensitivity of this species to monoterpene‐rich essential oils [34, 35].

The strong insecticidal activity of TP‐EO may be attributed to its major volatile constituents, including monoterpenes such as α‐pinene, (*E*)‐pinocarveol, (*E*)‐verbenol, verbenone, α‐campholenal, α‐terpineol and (*E*)‐carveol. These compounds are known to interfere with insect neural and respiratory function [36, 37]. The rapid decline in survival probability, coupled with low LC_50_ values, supports the EO's potential as a botanical insecticide.

TP‐EO exhibited notable insecticidal activity against stored‐product insects, with LC_50_ values of 5.446 µL/L for S. granarius and 8.171 µL/L for T. confusum. These results are comparable to those previously reported for botanical insecticides, such as Tetraclinis articulata essential oil, which showed LC_50_ values of 2.7 µL/L against S. granarius and 4.4 µL/L against T. confusum [18]. These results highlight the potential of TP‐EO as a natural alternative in the integrated pest management of stored grains.

### 2.4 Antibacterial Activity

In the present study, the antibacterial activity of TP‐EO was evaluated against *E. coli*, *K. pneumoniae*, *P. penneri*, *P. mirabilis*, *P. aeruginosa*, *A. baumannii*, *S. epidermidis*, *S. aureus*, *E. faecalis*, and *N. gonorrhoeae*. As shown in Table 4, the disc diffusion assay revealed that TP‐EO exhibited weak antibacterial activity against *Pr. mirabilis*, moderate activity against *E. coli* 1, *Escherichia coli* 2, *K. pneumoniae* 2, *P. aeruginosa*, *E. faecalis* 1, and *N. gonorrhoeae*, and strong activity against *P. aeruginosa*, *A. baumannii*, *S. epidermidis*, *S. non‐aureus*, and *S. aureus*.

**TABLE 4:** Antibacterial activity of *T. polium* EO.

Bacterial strains	IZD (mm)	MIC (µL/mL)	MBC (µL/mL)	MBC/MIC	Effect
*Escherichia coli* 1	13.0 ± 1.2	5 ± 0.1	10 ± 0.10	2	Bactericidal
*Escherichia coli* 2	10 ± 1.5	10 ± 0.1	20 ± 0.1	2	Bactericidal
*Klebsiella pneumoniae* 1	+	+	+	+	+
*Klebsiella pneumoniae* 2	11 ± 0.5	10 ± 0.0	20 ± 0.0	2	Bactericidal
*Proteus penneri*	+	+	+	+	+
*Proteus mirabilis*	8.2 ± 1.4	20 ± 0.1	40 ± 0.1	2	Bactericidal
*Pseudomonas aeruginosa* 1	11.0 ± 1.6	10 ± 0.1	20 ± 0.1	2	Bactericidal
*Pseudomonas aeruginosa* 2	15.5 ± 0.6	2.5 ± 0.1	5 ± 0.1	2	Bactericidal
*Acinetobacter baumannii*	20.5 ± 0.5	0.6 ± 0.1	0.6 ± 0.1	1	Bactericidal
*Staphylococcus epidermidis*	15.5 ± 1.4	2.5 ± 0.1	5 ± 0.1	2	Bactericidal
*Staphylococcus non aureus*	17 ± 0.5	1.25 ± 0.0	1.25 ± 0.0	1	Bactericidal
*Staphylococcus aureus*	30 ± 0.6	0.3 ± 0.0	0.3 ± 0.0	1	Bactericidal
*Enterococcus faecalis*	14 ± 0.8	2.5 ± 0.1	5 ± 0.2	2	Bactericidal
*Neisseria gonorrhoeae*	13 ± 1.5	5 ± 0.1	5 ± 0.1	1	Bactericidal

**(+)**: not active

Based on the microdilution assay, TP‐EO exhibited a bactericidal effect against all tested bacteria, except *Klebsiella pneumoniae* 1 and *Proteus penneri*, with MIC values ranging from 2.5 ± 0.1 to 20 ± 0.1 µL/mL. Furthermore, a previous study reported that the TP‐EO collected from the Midelt region (Morocco) showed weak antibacterial activity against *P. aeruginosa* (8 ± 0.4 mm), moderate activity against *E. coli* (10 ± 0.5 mm) and *K. pneumoniae* (13 ± 0.5 mm), strong activity against *S. aureus* (23 ± 1.4 mm), and *Acinetobacter baumannii* (15 ± 0.8 mm). The corresponding MIC values were 5.62 mg/mL for *P. aeruginosa*, 0.17 mg/mL for *S. aureus*, 2.81 mg/mL for *A. baumannii*, and 5.62 mg/mL for *E. coli* [21].

The antibacterial activity of TP‐EO was evaluated against clinical bacterial isolates obtained from different biological samples and previously characterized according to their antibiotic resistance profiles, including ESBL‐producing, methicillin‐resistant, penicillinase‐producing, and multidrug‐resistant strains. Interestingly, TP‐EO exhibited notable antibacterial activity against several resistant isolates, particularly S. aureus, with an inhibition zone diameter reaching 30 mm. These findings suggest that TP‐EO may represent a promising natural source of antimicrobial agents against resistant clinical pathogens.

The bactericidal antimicrobial activity of TP‐EO may be associated with the presence of major terpenoid constituents, such, β‐eudesmol, β‐pinene, α‐eudesmol acetate, and as α‐pinene, or by the synergistic and antagonistic effects between TP‐EO components [38].

## 3 Conclusions

The present study made it possible to characterize the chemical composition of *T polium* essential oil and to evaluate its main biological activities: Antimicrobial activity, antioxidant power, and insecticidal activity. Chromatographic analysis revealed a volatile fraction rich in monoterpenic and sesquiterpenic compounds that could explain the observed biological effects. In vitro tests show that, the essential oil exerts notable antimicrobial activity against several tested strains, moderate antioxidant potential in the tests used, and marked insecticidal action on the evaluated stages, suggesting a versatile spectrum of action. These results confirm that *T. polium* essential oil is a promising natural source of bioactive molecules of pharmaceutical and agronomic interest. Nevertheless, further investigations are needed to solidify these prospects: Fractionation studies and structural identification of the active components. In conclusion, *T polium* essential oil appears to be a promising natural candidate for antimicrobial, antioxidant, and insecticidal applications.

## 4 Experimental Section

### 4.1 Plant Material and Essential Oil Extraction

The aerials parts of *T. polium* were collected in May 2022 during the flowing stage from Ras El Ma (Qabouyawa) (35°08 ‘10 “N, 2°25’ 30” W) located in the region of Rif, in the northeast of Morocco. The plant species was identified by Professor Abdelillah Rahou, at the Laboratory of Plant Biotechnology and Molecular Biology, Faculty of Sciences of Meknes. A voucher specimen (No. 105502) has been deposited in the herbarium of Rabat, Morocco. The TP‐EO was extracted by using the hydro‐distillation method in a Clevenger‐type apparatus. During each test, 200 g of the dried aerials parts was treated. The extraction time was around 3 h. Three repetitions were performed. The EO was stored at 4°C in the dark until use.

### 4.2 Characterization of Essential Oil

#### 4.2.1 Samples and Chemicals

All solvents were purchased from Merck Life Science (Merck KGaA, Darmstadt, Germany). C_7_‐C_30_ saturated alkanes mixture (1000 µg/mL each component in hexane) (Merck Life Science) was utilized for the determination of linear retention indices (LRIs). For GC‐MS and GC‐FID analyses, distilled EO (10 µL) was diluted in 990 µL of *n*‐heptane (dil. 1:100).

#### 4.2.2 GC‐MS Analysis

Separation and identification of terpenes and terpenoids in TP‐EO leaves was performed on a system consisting of a gas chromatograph coupled with a single quadrupole mass spectrometer (GCMS‐QP2020, Shimadzu, Duisburg, Germany). The instrument was equipped with an AOC‐20i auto‐sampler and split‐splitless injector (280°C). The capillary column was a low‐polarity one, namely SLB‐5ms 30 m × 0.25 mm ID × 0.25 µm d*_f_* (Merck Life Science, Merck KGaA, Darmstadt, Germany). Temperature program: from 50° to 320°C at 3.0°C min^−1^. Injection volume and split ratio: 0.5 µL, split 1:10. Helium was used as carrier gas at an average linear velocity of 30 cm s^−1^ (inlet pressure 26.7 kPa). MS system operated in scan acquisition mode monitoring all fragment ions within a mass range of 40–550 *m/z*. Ion source temperature: 220°C; interface temperature: 250°C. The GCMS solution software (version 4.50 Shimadzu) was used for data collection and handling. The identity of each component was revealed through the use of two different identification parameters: Spectral match (≥ 85%) and LRI correspondence (±5). The FFNSC 4.0 mass spectral database (Shimadzu) was used for the identification of terpene and terpenoid compounds.

#### 4.2.3 GC‐FID Analysis

Quantitative analyses were carried by using a GC‐2010 instrument (Shimadzu) equipped with a flame ionization detector (FID). GC capillary column, temperature program and carrier gas were the same as described for the GC‐MS system, except for the initial inlet pressure of 99.5 kPa (the average linear velocity was 30 cm s^−1^). The temperature of FID was set at 300°C (sampling rate: 40 ms). FID gas flows: 40 mL/min for H_2_, 30 mL/min for the make‐up gas (N_2_) and 400 mL/min for air. Data were collected and processed using the LabSolution software (version 5.92, Shimadzu). Each sample was analyzed for three consecutive runs.

The identification of volatile compounds was based on comparing their mass spectra with those in the NIST library and comparing their retention indices (LRI) with data from the literature, mainly from Adams (2007) [39].

### 4.3 Antioxidant Activity

#### 4.3.1 DPPH Radical Scavenging Test

The radical scavenging capacity of DPPH (2,2‐Diphenyl‐1‐picrylhydrazyl) was evaluated according to the method described by Sadiki et al. [18]. Briefly, a volume of 1 mL of each concentration of EO dissolved in methanol (0.625, 1.25, and 2.5 µL/mL) was mixed with 1 mL of methanolic solution DPPH (0.04%). The mixtures were vortexed and stored for 30 min at room temperature in the dark. Then, the absorbance was measured with a spectrophotometer at 517 nm. Ascorbic acid and gallic acid were used as a standard. The percentage of DPPH radical scavenging was calculated according to the following formula:

$$100\times \left[\left(A_{0}-A_{t}\right)/A_{0}\right]$$

where, *A*_0_ is the absorbance of blank and *A*_t_ is the absorbance in the presence of EO.

#### 4.3.2 ABTS Radical Cation Scavenging Assay

The assay was performed as previously described by Sadiki et al. [18]. ABTS (2,2′ ‐Azino‐bis‐(3‐ethylbenzothiazoline‐6‐sulfonic acid), diammonium salt) radical cation (ABTS^+^) was generated by mixing together equal volumes of ABTS aqueous solution (7 mM) with potassium persulfate (2.45 mM). The mixture was left in the dark at room temperature for 12–16 h before use. Prior to testing, ABTS^+^ solution, was diluted with methanol to an absorbance of 0.7 ± 0.02 at 734 nm. 0.10 mL of each methanolic dilution of EO was mixed with 1.90 mL ABTS^+^ solution. The absorbance at 734 nm was measured after 6 min of reaction. ABTS^+^ scavenging capacity (%) was calculated using the formula:

$$100\times \left[\left(A_{\mathrm{control}}-A_{\mathrm{sample}}\right)/A_{\mathrm{control}}\right]$$

where, *A*_control_ is the absorbance of the control and *A*_sample_ is the absorbance in the presence of EO.

#### 4.3.3 Insecticidal Activity of Essential Oil

The insecticidal activity of TP‐EO against *S. granarius* (Curculionidae) and *T. confusum* (Tenebrionidae) was evaluated according to the method described by Sadiki et al. [18]. The insects originated from isolated strains of wheat kernels that have been attacked and spread by *S. granarius* and *T. confusum*. These strains were raised in the laboratory, in a well‐ventilated room, where the temperature varied between 24 and 28°C and humidity of 70%, on durum wheat, in a ventilated fabric bag. The EO fumigation was carried out in hermetic and transparent plastic boxes, with a capacity of 1L as an exposure chamber to test the toxicity of the EO against the adults of *S. granarius* and *T. confusum*, at a temperature ranging from 24 to 28°C and a humidity of 70%. In each box five Petri dishes were put, to ensure five repetitions. Each petri dish contains ten adult insects. The tests were carried out under the breeding conditions. The EO was spread on Whatman‐type filter paper which was placed inside the exposure chamber. Eight doses were applied, *viz*. 1.25, 2.5, 5, 7.5, 10, 12.5, 15, 17.5, and 20 µL, and an untreated batch served as a control. Mortality control was carried out by counting dead insects from the first day of treatment until the death of all individuals. Throughout the exposure period, the LC_50_ and LC_99_ doses were determined as well as the lethal times required for the death of 50% (LT_50_) and 99% (LT_99_) of adults exposed to different concentration levels of EO.

### 4.4 Antibacterial Activity

#### 4.4.1 Bacterial Strains and Growth Conditions

The bacterial strains used in this study were obtained from the Laboratory of Microbiology at Military Hospital Moulay Ismail, in Meknes, Morocco (Table 5). The antibacterial activity of the TP‐EO was assessed using fourteen clinical bacteria isolated from different sources Bacterial strains were cultured at 37°C for 24 h on Mueller Hinton agar (Merck Life Science, Merck KGaA, Darmstadt, Germany) medium. Then, bacterial suspensions were prepared in sterile distilled water and adjusted to the equivalent of 0.5 McFarland standard (10^8^ cfu/ mL).

**TABLE 5:** Profile of bacteria tested.

Bacterial strains	Gram	Profile	Sex	Type of sample
*Escherichia coli 1*	Gram negative bacilli	Cephalosporinase low level	Female	Cytobacteriological urine exam
*Escherichia coli 2*	Gram negative bacilli	ESBL	Male	Cytobacteriological urine exam
*Klebsiella pneumoniae 1*	Gram negative bacilli	ESBL	Male	Sonde urinaire
*Klebsiella pneumoniae 2*	Gram negative bacilli	High level penicillinase	Female	Cytobacteriological urine exam
*Proteus penneri*	Gram negative bacilli	High level penicillinase	Male	Pus
*Proteus mirabilis*	Gram negative bacilli	High level penicillinase	Male	Cytobacteriological urine exam
*Pseudomonas aeruginosa 1*	Gram negative bacilli	Bas niveau naturelle Cephalosporinase	Female	Coproculture (stool bacteria)
*Pseudomonas aeruginosa 2*	Gram negative bacilli	Cephalosporinase bas niveau FQ‐R	Male	Cytobacteriological urine exam
*Acinetobacter baumannii*	Gram negative bacilli	Multi‐resistant	Male	Cytobacteriological urine exam
*Staphylococcus epidermidis*	Gram positive cocci	Methicillin‐resistant	Male	Urethral
*Staphylococcus non aureus*	Gram positive cocci	Methicillin‐sensitive	Female	Pus
*Staphylococcus aureus*	Gram positive cocci	Methicillin‐sensitive, Penicillinase (+)	Male	Pus
*Enterococcus faecalis*	Gram positive cocci		Female	Cytobacteriological urine exam
*Neisseria gonorrhoeae*	Diplococcus (intra and extra cellular)	Wild Phenotype	Male	urethral

ESBL: extended‐spectrum beta‐lactamases.

#### 4.4.2 Disk Diffusion Method

The antibacterial activity of the TP‐EO was tested by determined disk diffusion method as described as by Sadiki et al. [18]. Well‐isolated colonies were transferred into tubes containing sterile distilled water in order to obtain microbial suspensions with a turbidity close to that of McFarland 0.5 (10^8^ CFU mL). Subsequently, the entire surface of the agar (Mueller Hinton agar for non‐fastidious bacteria; blood and chocolate agar for fastidious bacteria) was established by this microbial suspension. Afterward, the impregnated sterile filter disc with 10 µL of EO was placed on Petri dishes. After incubation of Petri dishes at 37°C for 24 h, the inhibition zones diameters (IZD) were measured in millimetre. The antibacterial activity was classified into tree levels: Strong (IZD > 15 mm), moderate (10 mm ≤ IZD ≤ 15 mm) and weak (IZD < 10 mm) (20).

#### 4.4.3 Broth Macrodilution Method (Non‐Fastidious Bacteria)

Minimum inhibitory and minimum bactericidal concentrations (MIC, MBC) were determined using a broth macro‐dilution method [18]. Briefly, a volume of 400 µL of EO were placed in a sterile tube containing 4.6 mL Mueller Hinton broth (MHB), supplemented with Tween 80 (0.01%, *v/v*). Then, a cascade dilution was prepared to obtain a final concentration of 80, 40, 20, 10, 5, 2.5, 1.25, 0.6, and 0.3 µL/mL. 13 µL of a bacterial inoculum, were deposited in each of the tubes of the range. A control of bacterial growth was also carried out, for which 13 µL of the standardized inoculum were placed in MHB‐Tween 80 (0.01%, *v/v*). Then, after incubation at 37°C for 24 h, the tubes were centrifuged at 5.000 x g, for 5 min, at 20°C. The MIC was determined from the lowest concentration of EO showed no visible bacterial growth with naked eye. The MBC was the lowest concentration of EO, which shows no bacterial growth. The tubes showing no visible growth with the naked eye (From MIC) and the control tube were streaked on Petri dishes containing MHA and incubated at 37°C for 24 h. Furthermore, the MBC/MIC was calculated as follows: If MBC/MIC ≤ 4, the effect is bactericidal, if MBC/MIC > 4, the effect is bacteriostatic. All the experiments were carried out in triplicate.

#### 4.4.4 Agar Dilution Method (Fastidious Bacteria)

Essential oil was incorporated in a medium (Blood Agar for *E. faecalis* and chocolate Agar *N. gonorrhoeae*) 2% Tween 80, and cooled to obtain dilutions of 80 to 0.3 µL/mL in a final volume of 20 mL in the Petri dishes. Controls containing only 2% Tween 80 medium without EO were also performed to demonstrate the absence of antibacterial activity of Tween [18]. The incubation time was 24 h at 37°C. The minimum inhibitory concentration (MIC) corresponds to the lowest concentration of essential oil that inhibits any culture visible to the naked eye after the culture time specified for each isolate. Samples were taken from the control tubes and from each of the tubes devoid of bacterial pellet and then streaked onto the agar. The seeded dishes were incubated for 24 h at 37°C. The CMB was deduced from the first dish devoid of bacteria.

### 4.5 Statistical Analysis

The data are expressed as means ± SD of the biological replicates. Lethal concentrations (LC_50_ and LC_99_) of the EO were determined using logistic regression in dose‐response assays based on the concentration Probit‐mortality according to Finney [31] and mortalities were corrected by the Abbott formula [40]. Statistical analyses were conducted using the SPSS software for Windows (Version 22).

## Author Contributions

**Fatima Zahra Sadiki**: conceptualization, methodology, investigation, data curation, and writing – original draft. **Souhail Channaoui**: investigation, data curation. **Aziz Bouymajane**: data curation, investigation. **Mostafa El Idrissi**: investigation, data curation. **Ali Amechrouq**: investigation, data curation. **Mohammed Sbiti**: investigation, data curation. **Francesco Cacciola**: supervision, writing – review and editing, and project administration. **Mohamed Chabbif**: conceptualization, supervision, and writing – review and editing. All authors have read and agreed to the published version of the manuscript.

## Conflicts of Interest

The authors declare no conflicts of interest.

## Data Availability

The data that support the findings of this study are available from the corresponding author upon reasonable request.
